# The Anti-Obesogenic Effects of Muscadine Grapes through Ciliary Neurotrophic Factor Receptor (Cntfr) and Histamine Receptor H1 (Hrh1) Genes in 3T3-L1 Differentiated Mouse Cells

**DOI:** 10.3390/nu16121817

**Published:** 2024-06-09

**Authors:** Samia S. Messeha, Meenakshi Agarwal, Sherif G. Gendy, Sheikh B. Mehboob, Karam F. A. Soliman

**Affiliations:** 1 College of Science and Technology, Florida A&M University, Tallahassee, FL 32307, USA; samia.messeha@famu.edu; 2 College of Pharmacy and Pharmaceutical Sciences, Institute of Public Health, Florida A&M University, New Pharmacy Building, 1415 ML King Blvd., Tallahassee, FL 32307, USA; 3 Center for Viticulture & Small Fruit Research, Florida A&M University, Tallahassee, FL 32317, USA; meenakshi.agarawal@famu.edu; 4 School of Allied Health Sciences, Florida A&M University, Tallahassee, FL 32307, USA; sherif.gendy@famu.edu

**Keywords:** muscadine grape extract, 3T3-L1, Preadipocytes, adipocytes, obesity, gene expression

## Abstract

Obesity and type 2 diabetes are prevalent metabolic diseases that have significant links to several chronic diseases, including cancer, diabetes, hypertension, and cardiovascular disease. Muscadine grape extracts have shown the potential to reduce adiposity and improve insulin sensitivity and glucose control. Thus, this study was designed to determine the potential of muscadine grape berries extract (Pineapple and Southern Home) for its antiobesity properties in 3T3-L1 cells as a model for obesity research. The current study’s data indicated the total phenolic content (TPC) and 2,2-diphenyl-1-picrylhydraziyl (DPPH) activity were higher in cultivar (CV) Southern Home, meanwhile, elevated the total flavonoid content (TFC) in Pineapple. Both extracts were safe across the tested range (0–5 mg/mL). A noticeable reduction in lipid accumulation was also found in extract-treated cells. In preadipocytes and adipocytes, the tested extracts showed significant alterations in various genes involved in glucose homeostasis and obesity. The most remarkable findings of the current study are the upregulation of two genes, Cntfr (+712.715-fold) and Hrh1 (+270.11-fold) in CV Pineapple extract-treated adipocytes 3T3-L1 and the high fold increase in Ramp3 induced by both Pineapple and Southern Home in pre-adipose cells. Furthermore, the tested extracts showed a potential to alter the mRNA of various genes, including Zfp91, B2m, Nr3c1, Insr, Atrn, Il6ra, Hsp90ab1, Sort1, and Npy1r. In conclusion, the data generated from the current study suggested that the two extracts under investigation are considered potential candidates for controlling insulin levels and managing obesity.

## 1. Introduction

Obesity is classified as either a multifaceted disease or a non-communicable condition. This condition is characterized by excessive fat accumulation in adipocytes, leading to high lipids (triglycerides) levels in the plasma and tissues such as the liver and muscles. Various subsets of neurons interact with peripheral nutrients and hormones to control an organism’s energy balance and body weight. Obesity occurs when energy intake and expenditure are out of balance, resulting in a variety of physiological abnormalities and chronic diseases such as hypertension, heart disease, osteoarthritis, cardiovascular disease, type II diabetes mellitus, malignancies, reproductive hormone abnormalities, and respiratory problems, among others [1,2,3,4].

Multiple environmental variables, including a sedentary lifestyle and genetic vulnerability, contribute to the increased risk of obesity [5,6]. Due to its alarming pace of expansion, it has garnered significant attention as a possible global health threat [7,8,9,10]. As a result, it is critical to address the consequences of obesity and take initiative-taking measures to prevent it. A variety of public health intervention programs and initiatives have been proposed as solutions to the growing obesity epidemic. These include changes to dietary habits, exercise levels, lifestyle choices, pharmacological treatment, surgical weight loss, etc. [11,12]. Despite this, the outcomes of these initiatives have not been very fruitful in addressing the increasing incidence of obesity and type 2 diabetes [13].

Furthermore, there are certain disadvantages associated with the use of anti-obesity medications, including the potential for encountering side effects and the likelihood of weight regain after discontinuing the prescription [14]. Therefore, it is vital to delve into new research studies on healthy meals or pharmaceuticals without damaging side effects or undesirable consequences and comprehend their influence on controlling obesity. 3T3-L1 preadipocytes are often employed in in vitro lipid metabolism and adipogenesis investigations. Under certain conditions, these cells can transform into a phenotype similar to adipocytes by changing gene expression. [15,16,17]. Various dietary polyphenols from plant sources rich in bioactive compounds have been reported to reduce obesity through different mechanisms. These include preventing adipocyte hypertrophy, inhibiting preadipocyte differentiation, increasing lipolysis, or induction of fat cell apoptosis [18,19]. For example, a previous study by Song et al. showed treating 3T3-L1 cells with blueberry peel extract could lower the lipid accumulation and adipocyte differentiation of 3T3-L1 cells [20]. Another study by Kubota et al. showed that *Blumea balsamifera*, a medicinal plant extract, causes a significant decrease in the activity of glycerol-3-phosphate dehydrogenase in 3T3-L1 preadipocytes suggesting its potential role in inhibiting adipogenesis [21].

From this perspective, fruits like grapes are regarded as a rich source of phytochemicals that contain vitamins, minerals, fibers, phenolic acids, flavanols, etc. Muscadine grapes (*Vitis rotundifolia*), which are commonly grown in the southeastern United States, are exceptionally high in polyphenolic compounds such as ellagic acid, gallic acid, procyanidin B2, catechin, and catechin gallate, quercetin, cyanidin, and delphinidin, among others [22,23,24]. Muscadine grapes exhibit a variety of hues, including bronze, red, purple, and black. In addition, Muscadine grapes differ from bunch grapes (*Vitis vinifera*) in terms of phytochemical composition, with more antioxidant compounds, the presence of anthocyanin 3,5-glucosides in purple cultivar skin, a high concentration of ellagitannins and ellagic acids in seeds and skin [23,25]. To date, no research has been conducted to examine the impact of muscadine grape phytochemicals on the differentiation of adipocytes from 3T3-L1 cells. A couple of studies have shown that muscadine grape may help reduce adiposity. One research group found that phytochemicals from muscadine cultivar (CV) Noble improved insulin sensitivity and glucose control in C57BL/6J mice [26]. Another recent study found that adding muscadine grape extract and probiotics to a Western diet lowered obesity in adult female C57BL/6 mice [27]. However, the molecular process remains unknown, including how phytochemicals from muscadine grape lower adiposity and how transcription changes occur in preadipocyte cells.

This study investigates the potential of Muscadine grape berry extract (MGE) from phytochemical-rich muscadine grapes in regulating obesity. We utilized whole berry extract from bronze- and purple-colored berries of muscadine cultivars to investigate the impact on 3T3-L1 preadipocyte cells and adipocyte cell differentiation at the molecular and cellular levels. Our research would help to build vital nutraceutical food supplements to broaden their application beyond fresh fruit and wine.

## 2. Materials and Methods

### 2.1. Berry Sample Collection

Berries from two separate muscadine grape cultivars having bronze and deep purple-black berries were harvested from the vineyard at the Center for Viticulture and Small Fruit Research, Florida Agriculture and Mechanical University, Tallahassee, FL, USA, during the September 2021 vintage. Berries were immediately preserved on ice before being transported to the laboratory and processed for extraction.

### 2.2. Muscadine Grape Extract Preparation

Ten grams of berries were crushed, mixed with 7 mL of methanol, and homogenized for 10 min. The suspension was spun at 14,000 rpm for 15 min. As stated above, the supernatant was carefully removed, and the remaining residues were extracted again with 3 mL of methanol. The combined organic solvent extracts were vacuum-dried in a concentrator at room temperature in the dark. The dried materials were then redissolved in dimethyl sulfoxide (DMSO) at 150 mg/mL concentration level for bioactivity assessment.

### 2.3. Total Phenolic Content (TPC) Measurement

The TPC of each extract was measured using the Folin–Ciocalteu (FC) assay, following the method reported by Singleton and Rossi with certain modifications [28]. In summary, a 100 µL sample of grape berry methanolic extract (diluted 1:5) was combined with 150 µL of the FC reagent (diluted 1:1) and incubated for 5 min at room temperature. Afterward, 0.5 mL of a solution containing 20% sodium carbonate was added. The suspension was then kept in a dark and incubated for an additional 40 min at 37 °C. The absorbance was measured at a wavelength of 765 nm. TPC was quantified as milligrams per gram of dry weight of the original sample. Gallic acid was used as the phenolic standard, with 10 to 100 µg/mL concentrations. Analysis was done in triplicates.

### 2.4. Flavonoid Measurement

Flavonoid measurements were performed as previously described with some modifications [28]. Briefly, 300 µL of extract was mixed with 300 µL of 5% sodium nitrite solution and incubated for 5 min. After that, 3 mL of 10% aluminum chloride was added and incubated for 6 min. The suspension was mixed well with 2 mL of 1 M sodium hydroxide and 3.3 mL of distilled water. Absorbance was measured at 510 nm using a blank as a reference. Catechin was used as a standard for the calibration curve. The total flavonoid content of the extract was expressed as mg catechin equivalents per gram of sample (mg/g).

### 2.5. DPPH Activity

The antioxidant capacity of the berry extracts was determined by measuring their ability to scavenge free radicals using the 2,2-diphenyl-1-picrylhydraziyl (DPPH) compound, as described in a previous study [29]. Briefly, 50 µL of the extract was mixed with 2 mL of freshly prepared DPPH methanolic solution (60 μM). The mixture was then incubated for 30 min in the dark at room temperature. The absorbance was measured at 515 nm. DMSO was used as a control instead of as a sample. The blank was prepared with the methanolic dilution of DPPH. Data were calculated as the percentage scavenging of DPPH radical and calculated using the following equation: DPPH%= [1 − (A sample − A blank)/(A control − A blank)] × 100.

### 2.6. Cell Culture and Adipocyte Differentiation

The 3T3-L1 cells were acquired from the ATCC and subjected to differentiation using a previously established protocol [29] with some modifications. The cells were incubated in Dulbecco’s modified eagle medium (DMEM) supplemented with 10% fetal bovine serum (FBS) and 100 µg/mL penicillin-streptomycin at a temperature of 37 °C in a 5% CO_2_ for 2 days or until they reached 70% confluency. The media was substituted with a differentiation medium comprising 90% DMEM, 10% FBS, 1.0 µM dexamethasone, 0.5 mM 3-isobutyl-1-methylxanthine (IBMX), and 1.0 µg/mL insulin. After 2 days, the medium was substituted with DMEM solution containing 1.0 µg/mL insulin every 2 days and left to incubate for 8 days or until the development of lipid droplets occurred.

### 2.7. Oil Red O Staining

The oil red O stain kit (Sigma, St. Louis, MO, USA) was used following the manufacturer’s instructions. In short, the medium was gently removed and rinsed twice with phosphate-buffered saline. The cells were fixed with 10% formalin for 40 min. Cells were rinsed twice with water, then 60% isopropanol was added and incubated for 5 min. Isopropanol was discarded and replaced with Oil Red O solution to cover the cell surface and incubate for 10 min. The cells were rinsed four times with sterile water before Hematoxylin was added and incubated for one minute. The cells were rinsed four times with distilled water. The cell surface was coated with water and examined under a microscope. The microscopic images were captured using an inverted EVOS M5000 microscope (Thermo Fisher Scientific, Carlsbad, CA, USA) with a 20× objective lens as described previously [30]. The images were processed using Fiji ImageJ software 2.1.0.

### 2.8. Cytotoxicity Assay

The cytotoxic effect of the two extracts under investigation (Southern Home and Pineapple) in 3T3-L1 cells was determined using Alamar Blue^®^. Cells were first plated in 96-well plates at a density of 5 × 105 cells/well and placed in the CCI under the previously mentioned settings. Next, cells were treated with the extracts at 0 to 5 mg/mL concentrations. Control cells were exposed to DMSO, and Blank wells with only media/media with extracts were used. The experiments were performed in triplicates. After 24 h exposure period, 20 µL Alamar Blue^®^ (0.5 mg/mL) was added to each well, and the plates kept in CCI for 4 h. Lastly, we read the plates at 530/590 nm (excitation/emission wavelength) using a Synergy H.T.X. multi-mode microplate reader (BioTek Instruments, Inc., Winooski, VT, USA). Data were analyzed using Excel software(Microsoft 365, 2019) and GraphPad Prism 6.2 software (GraphPad Software Inc., San Diego, CA, USA).

### 2.9. Gene Expression Analysis

#### 2.9.1. Treating Cells

According to the data obtained from the cytotoxicity study, we first selected the safe concentration of 2.4 mg/mL that does not interfere with the cell viability. The impact of Southern Home and Pineapple extracts on different genes mediating obesity was established in a T-25 flask loaded with 5 × 10^5^ cells. We treated 3T3-L1 cells in the pre-adipose stage and during the differentiation period. By the end of each experiment, cells were collected and centrifuged to obtain the pellets.

#### 2.9.2. RNA Extraction and Complementary DNA (cDNA) Synthesis

Extracting the total RNA from 3T3-L1 cell pellets was established as follows: homogenize the pellets with TRIzol reagent (Life Technologies, Paisley, UK), separating the RNA-rich layer using chloroform, pellet the RNA with isopropyl alcohol, then wash the RNA pellets in 75% ethanol solution before dissolving it in the nuclease-free water. The concentration of RNA was measured in each sample. We followed the manufacture protocol for the DNA-free™ kit (Thermo Fisher Scientific) to eliminate the genomic DNA contamination. In contrast, the iScript™ cDNA Synthesis kit (Bio-Rad Laboratories, Hercules, CA, USA) was used to convert 1 μg of RNA into cDNA. The R.T. reaction started at 46 °C for 20 min, followed by 1 min. inactivation at 95 °C, using the CFX96 Touch Real-Time PCR Detection System (Bio-Rad). The obtained cDNAs were stored in a −80 °C freezer.

#### 2.9.3. Quantitative Reverse Transcription Polymerase Chain Reaction (qRT PCR) Obesity Array

A 96-well human obesity array (SAB Target List, cat # 10034386, Bio-Rad) was loaded with diluted cDNA (2.3 ng) and Sso Advanced™ Universal SYBR^®^ Green Supermix (Bio-Rad) for a final volume of 20 µL/well. The fluorescence quantification was accomplished using the Bio-Rad CFX96 Real-Time System (Bio-Rad). cDNA was amplified by 39 cycles: activation, denaturation, and annealing [31,32]. The qRT-PCR data were confirmed using at least three independent experiments.

### 2.10. Statistical Analysis

Data obtained from this study were analyzed using GraphPad Prism 6.2 software. Data are expressed as mean ± S.E.M. from three biological replicates. One-way analysis of variance was used to compare more than two groups (control vs. treated). CFX 3.1 Manager software (Bio-Rad Laboratories, Hercules, CA, USA) was used to quantify gene expression in the obesity arrays. The significance of differences was generated by analysis of variance (ANOVA) followed by Bonferroni’s multiple comparison test. Unpaired Student’s t-test was used to analyze two datasets. Differences were considered significant at *p* < 0.05 (as mentioned in the figures and legends).

## 3. Results

### 3.1. Total Phenolic, Flavonoid, and Antioxidant Capacity of MGEs

**Figure 1:**
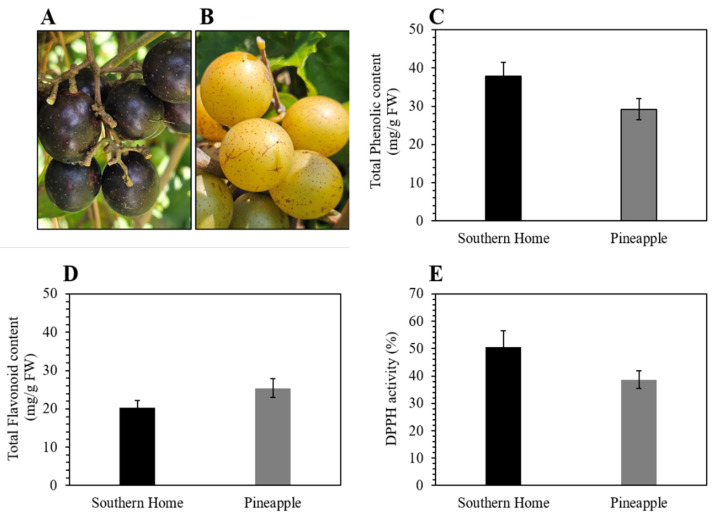
Characteristics of muscadine grapes. (**A**) Images of Berries from Southern Home. (**B**) Berries from Pineapple. (**C**) Assessment of TPC. (**D**) Assessment of TFC. (**E**) Assessment of DPPH activity. The error bar indicates the standard deviation from three triplicates.

An analysis was conducted on the ripe berries of muscadine cultivars, Southern Home and Pineapple, to determine their total phenolic content (TPC), total flavonoid content (TFC), and antioxidant levels. Figure 1 demonstrates that the TPC and DPPH activities of CV Southern Home were higher than in Pineapple, whereas the flavonoid content was lower in CV Southern Home as compared to CV Pineapple.

### 3.2. Cytotoxic Effects of Southern Home and Pineapple Extracts

**Figure 2:**
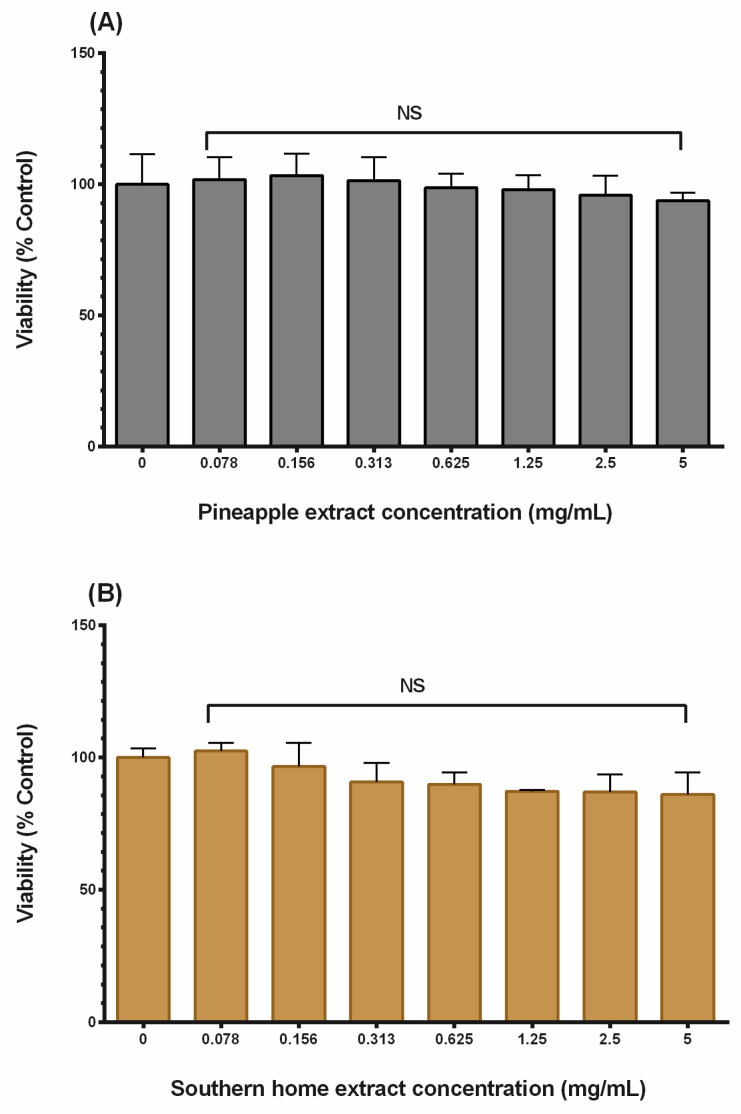
Cytotoxic effect of the examined extracts on 3T3-L1 cells. (**A**) Pineapple extract and (**B**) Southern Home extract. The cells under investigation were seeded in 96-well plates (5 × 10^5^ cells/well) and placed overnight in the cell culture incubator. They were exposed to different concentrations of each extract (0–5 mg/mL) and re-incubated for 24 h. The figures demonstrated the cell viability as a percentage of living cells compared to the control. The presented data were generated from three independent studies. The data were analyzed using one-way ANOVA followed by Bonferroni’s multiple comparisons test. N.S., nonsignificant.

The cytotoxic effects of the extracts in the 3T3-L1 model cell were examined using AB^®^ assays to detect metabolically active cells. Figure 2A,B showed a non-significant effect between control and cells treated at different concentrations (0–5 mg/mL) following 24-h exposure to the tested extracts.

### 3.3. MGEs Reduce Lipid Accumulation in Adipocyte Cells

**Figure 3:**
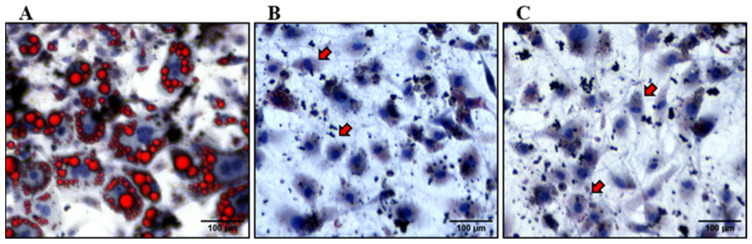
Analysis of lipid accumulation using oil red O staining. (**A**) Control cells without any treatment of MGE. (**B**) Treatment with MGE, Southern Home. (**C**) Treatment with MGE, Pineapple. Red stain indicates lipid droplets, whereas blue stain indicates the nuclei. The red arrows indicate the absence of lipid droplets in treated cells. Images were obtained using an inverted microscope at 60× magnification. Scale bar—100 µm.

To assess the impact of MGEs on lipid accumulation in differentiated adipocytes, oil red staining was conducted at eight days following the induction of differentiation. The control cells (absence of MGE treatment) showed the accumulation of lipid droplets upon differentiation into adipocytes, as shown in Figure 3A. In contrast, cells exposed to MGEs derived from Southern Home and Pineapple cultivars at a concentration of 2.4 mg/mL exhibited a significantly diminished accumulation of lipids (Figure 3B,C).

### 3.4. Impact of Extracts on the Expression of Obesity-Associated Genes

**Figure 4:**
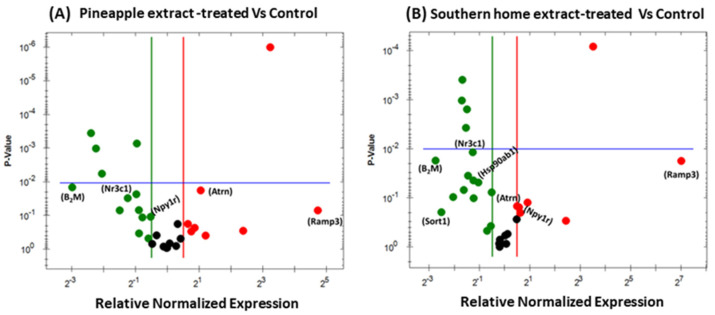

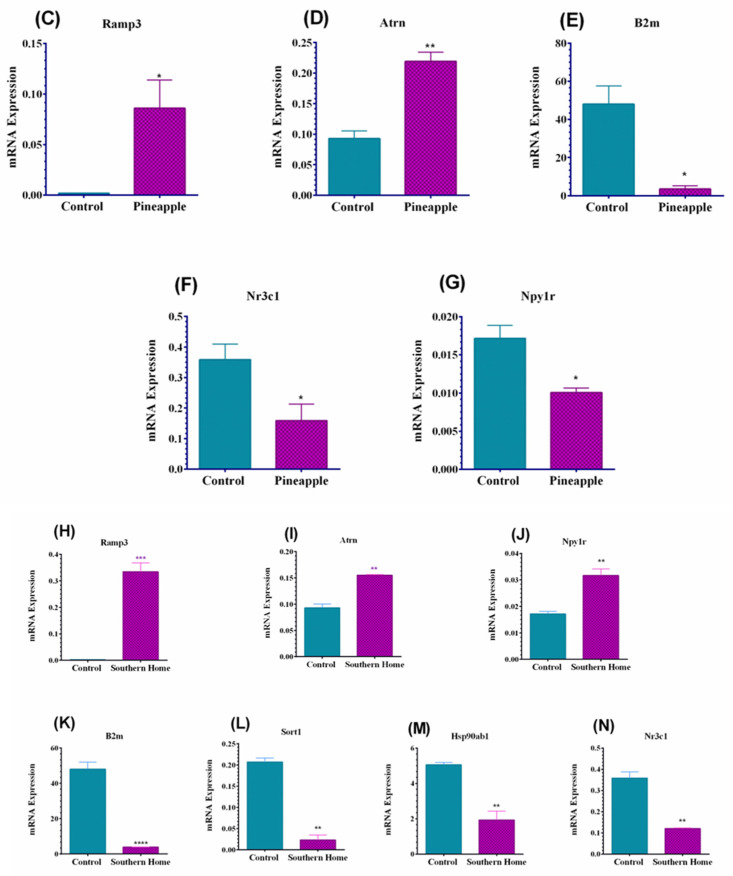
Categorization of obesity-related gene expression induced by Pineapple and Southern Home in pre-adipose 3T3-L1 cells. A volcano plot elucidates the altered genes in pre-adipose 3T3-L1 cells following 24-h exposure to 2.4 mg/mL of Pineapple (**A**) and Southern Home (**B**) extracts. The red dots refer to upregulated mRNAs, green dots for the inhibited genes, and black dots for unchanged. (**C**–**G**) the altered genes in Pineapple-treated cells. (**H**–**N**) the altered genes in Southern Home-treated cells. * *p* < 0.05, ** *p* < 0.01, *** *p* < 0.001, **** *p* < 0.0001.

RT-PCR was performed to test the underlying molecular mechanisms impacted by Pineapple and Southern Home in 3T3-L1 mouse cells. A list of obesity-linked genes was investigated using the 96-well plate obesity array. Following a previously described protocol, cells were treated for 24 h with a specific dose of the extracts equal to 2.4 mg/mL. An overview of the normalized obesity-related gene expression provided insight into the impact of both extracts on various genes involved in obesity. In Figure 4A,B, the red dots indicate the upregulated genes in both cell lines, whereas the green-colored dots represent the downregulated genes. The black dot in the middle panel indicates unchanged gene expression in both cell lines.

Interestingly, some genes were expressed in extremely low concentrations and were not shown in the generated data. Treating pre-adipose 3T3-L1 cells with the two extracts under investigation showed a significant alteration in crucial genes controlling insulin level and glucose homeostasis, as shown in Figure 4C–G for Pineapple-treated, Figure 4H–N for Southern Home-treated cells, as well as in Table 1. In brief, the obtained data showed a similar response pattern in 3T3-L1 cells. However, the most valuable finding is the outstanding upregulation of Receptor activity modifying proteins (Ramp3) in Southern Home-treated cells (+174.24-fold, Figure 4H) vs. +44.87-fold in Pineapple-treated cells (Figure 4C). An almost 2-fold increase in Attractin (Atrn) was induced by both extracts (Figure 4D,I and Table 1). On the other hand, significant inhibition of two genes, Beta-2-microglobulin; B2m (−13.59 vs. −12.20, Figure 4E,K, and Table 1) and the glucocorticoid receptor nuclear receptor subfamily 3, group C, member 1; Nr3c1 (−2.25 vs. −2.98, Figure 4F,N, and Table 1) was induced by Pineapple and Southern Home, respectively. An exclusive downregulation in sortilin1; Sort1 (−9.00-fold, Figure 4L and Table 1) and Heat shock protein 90kDa alpha family Class B member 1; Hsp90ab1 (−2.62-fold, Figure 4M and Table 1) was induced by Southern Home extract. Interestingly, an inverted alteration was found in the Neuropeptide Y1 receptor, Npy1r mRNA. Meanwhile, a significant attenuation was detected in Pineapple-treated cells (−1.70-fold, Figure 4G and Table 1), and an almost 2-fold increase in the genes was measured in Southern Home-treated cells (Figure 4J and Table 1).

**Figure 5:**
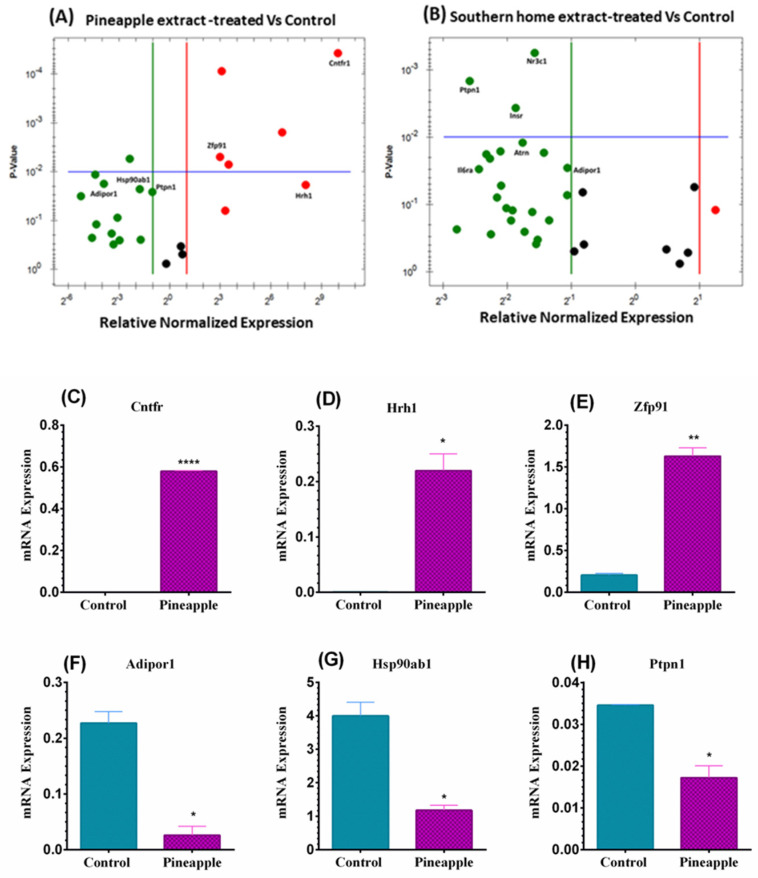

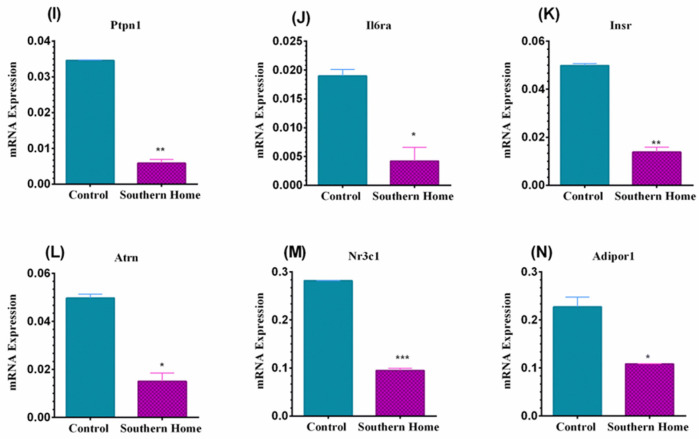
Categorization of obesity-related gene expression induced by Pineapple and Southern Home in differentiated 3T3-L1 cells. A volcano plot elucidates the altered genes in differentiated 3T3-L1 cells following exposure to 2.4 mg/mL of Pineapple (**A**) and Southern Home (**B**) extracts. The red dots refer to upregulated mRNAs, green dots for the inhibited genes, and black dots for unchanged. (**C**–**H**) the altered genes in Pineapple-treated cells. (**I**–**N**) the altered genes in Southern Home-treated cells. * *p* < 0.05, ** *p* < 0.01, *** *p* < 0.001, **** *p* < 0.0001.

On the other hand, incubating 3T3-L1 cells with the extracts during the differentiation phase showed an alteration in many genes other than Nr3c1 and Hsp90ab1 (Figure 5A–N and Table 2). At first glance, the Pineapple extract augmented the expression of three genes (Figure 5A), giving the remarkable upregulation in the ciliary neurotrophic factor receptor; Cntfr (+712.72-fold), followed by Histamine Receptor H1; Hrh1 (+270.11-fold), and Zinc Finger Protein 91; Zfp91 was the least (8-fold) as indicated in Figure 5C–E, respectively and Table 2. In contrast, we did not detect any gene upregulation in Southern Home-treated cells (Figure 5B).

Consistent with preadipose, the Southern Home extract showed the exact fold change in Nr3c1 (~−3-fold, Figure 5M and Table 2). Meanwhile, Hsp90ab1 was inhibited only by Pineapple extract (−3.40-fold, Figure 5G and Table 2). Furthermore, a significant downregulation in two more genes; adiponectin receptor protein 1; Adipor1 (−9.58-fold vs. −2.10-fold, Figure 5F and Figure 5N, respectively and Table 2), and the protein tyrosine phosphatase non-receptor type 1; Ptpn1 (−2.01-fold vs. −5.90-fold, Figure 5H and Figure 5I, respectively and Table 2) was measured in Pineapple and Southern Home extracts-treated cells, respectively.

Compared with preadipocytes, Southern Home extract showed an inverted effect in adipocytes 3T3-L1, inducing more than 3-fold inhibition in the Atrn gene. Furthermore, the mRNAs were also attenuated for two more genes, Interleukin 6 (Il-6) receptor alpha (Il6ra) and the insulin receptor (Insr), by Southern Home extract (Figure 5J,K, and Table 2), giving the highest fold change (−4.50-fold) in Il6ra (Figure 5J and Table 2).

## 4. Discussion

Muscadine grapes are valued for their bioactive metabolites and are reported to contain antioxidant, anti-inflammatory, and anticancer activities. In this study, we aim to explore the potential of muscadine grape extracts in regulating obesity by monitoring its impact on 3T3-L1 cells. We employed two muscadine cultivars: Pineapple, which has bronze berries, and Southern Home, which has purple berries. Since metabolites are essential for the nutritional value of grapes, we measured the TPC, TFC, and antioxidant activity of whole muscadine berry extracts. We found a higher level of TPC and antioxidants in CV Southern Home compared to the Pineapple. At the same time, the TFC was found to be higher in CV Pineapple. These results align with past findings that different muscadine cultivars had varying metabolite contents [33,34].

The TPC, TFC, and antioxidant activity results in our study are consistent with the range reported in previous research on different muscadine grape genotypes [35]. Molecules with antioxidant properties can neutralize ROS and mitigate oxidative stress, counteracting its deleterious effects associated with obesity [36]. A recent review [37] highlights that oxidative stress can be both a primary cause and a secondary effect of various disorders, including those related to obesity.

Our study elucidates the phenolic and flavonoid properties, drawing from insights from prior investigations. Extensive literature underscores the pivotal role of (poly)phenols and flavonoids in mitigating the risk of obesity and conferring beneficial effects under obese conditions [38]. These compounds have been associated with modulating various cellular responses, including alleviating intracellular oxidative stress, attenuating chronic low-grade inflammation, inhibiting adipogenesis and lipogenesis, and suppressing preadipocyte differentiation into mature adipocytes [39]. We selected muscadine cultivars, known for their elevated total stilbene content, including t-Piceid, t-Resveratrol, ε-Viniferin, and t-Pterostilbene, as demonstrated in our previous investigation [40,41]. The extraction method employed in our study has been tailored to efficiently capture the predominant phenolic and flavonoid constituents present in muscadine grape berries. Additionally, our previous study showed more stilbene in Southern Home than in Pineapple [40]. While both MGEs exhibited variations in their TPC, TFC, and antioxidant activity, no significant difference was observed in the lipid accumulation in 3T3-L1 cells following treatment, as observed by Oil red staining (Figure 3). This finding suggests that the phytochemical content found in each cultivar may be adequate to inhibit lipid accumulation at the measured concentration.

It is important to highlight that this is our first investigation utilizing whole muscadine grape berry extract and examining its impact on the 3T3-L1 cell. The utilization of the entire berry dilutes the total phytochemical content compared to extracts obtained from specific tissue or parts. The protocol used for preparing the extract is also a determinant factor for the concentration used. In the preliminary viability study, we began with low extract concentrations. However, we observed no effect on either cell viability in preadipocytes or lipid accumulation in adipocytes at these lower concentrations. Consequently, we tested a range of extract concentrations until we observed an effect on cell viability in preadipocyte cells. We selected a concentration from this range that maintained more than 80% cell viability, as depicted in Figure 3, while ensuring that it contained sufficient phytochemicals to exert its effect.

Emphasizing the molecular basis of obesity is an important tool in developing promising therapeutic agents against increased body weight. Therefore, in the current study, profiling the mRNA of various genes-mediating obesity was established for two sets (pre-adipose and differentiated 3T3-L1 mouse cells), following treatment with safe concentrations of the extracts under investigation (Figure 2A,B). Collectively, treating the 3T3-L1 cells at different phases showed interesting data. Meanwhile, a consistent alteration in some genes was measured at both phases, and each extract also induced an exclusive response. These genes directly regulate energy homeostasis or encode the hormones and receptors of anorectic and orexigenic peptides.

The most valuable outcome of this study is the outstanding upregulation of two genes, Cntfr and Hrh1, in Pineapple extract-treated differentiated 3T3-L1 cells, in addition to the high fold increase in Ramp3 that was induced by both extracts in pre-adipose cells. Indeed, a highly significant upregulation (+712.715) of Cntfr was measured in pineapple-treated -adipocyte cells. This receptor is a crucial activator of Cntfr, a member of the interleukin (IL)-6 type cytokine family [42,43]. Individuals with multiple sclerosis and amyotrophic lateral sclerosis experienced a marked decrease in weight when they received Cntfr [44]. Activation of leptin-independent or leptin-like intracellular signaling pathways is employed by Cntfr, as indicated by previous studies [45,46,47]. Also, a +270.11-fold increase in the Hrh1 gene was exhibited by pineapple-treated adipocyte cells. Hrh1 is one of the genes that influence both second-generation antipsychotics (SGAP) and mood stabilizers (MS) weight gain [48]. A previous study has demonstrated a significant association between the Hrh1 gene variants rs346070 and rs346074 and B.M.I. in Caucasian patients with a psychotic disorder [49]. The upregulated level of Zfp91 suggests the promising effect of Pineapple extract in managing obesity. The obtained data agree with previous studies that revealed the negative correlation between Zfp91 mRNA expression levels and body mass index (B.M.I.) [50].

Both CV Pineapple and Southern Home induced an outstanding upregulation of the gene Ramp3 in pre-adipose cells. This finding highlighted the importance of these extracts as a preventive approach, particularly the Southern Home extract that induced +174.24-fold vs. + 44.87-fold in Pineapple-treated cells. Ramps are an important family of G protein-coupled receptors (Gpcrs) accessory proteins [51,52] that initiate intracellular signaling [53]. The Ramps family contains three distinct transmembrane-spanning proteins; one is Ramp3, and the other is a receptor (G protein-coupled) activity-modifying protein 3. Postmenopausal obesity is linked to estrogen deficiency, which is accompanied by the accumulation of abdominal fat and decreased energy expenditure [54]. A previous study highlighted the relationship between estrogen and Ramp3 [55]. Knockout of Ramp3 in ovariectomized mice showed elevated serum insulin levels accompanied by exacerbation in obesity, adipocyte hypertrophy, and adipose tissue weight gain [55]. In pre-adipose cells, Pineapple and Southern Home extracts also showed an almost 2-fold increase in Atrn mRNA, whereas the same gene was inhibited in Southern Home-treated adipocytes. A previous in vitro study demonstrated that the absence of Atrn suppresses agouti (Agrp)-induced obesity [56]. Reduced body weight and adiposity, as well as partial suppression of diet-induced obesity, was also found in Atrn- mutant mice [57,58]. The obtained reduction of Atrn expression in Southern Home extract-treated adipocyte cells suggested that targeting Atrn could be a promising mechanism for managing obesity. Treating pre-adipose cells with either Pineapple or Southern Home extracts showed a reduction in Adipor1 and Ptpn1 gene expression. Downregulating Adipor1 demonstrated the value of these extracts in managing obesity, particularly Pineapple, which inhibited the gene by 10-fold vs. −2.10-fold in Southern Home-treated adipocyte cells. Adipor1 is involved in glucose and lipid metabolism. Indeed, an upregulated level of Adipor1 was found in obese subjects that could be related to metabolic disorders [59]. Furthermore, Adipor1 gene expression in chondrocytes positively correlates with a patient’s body mass index (B.M.I.) [60].

On the other hand, Ptpn1is expressed in multiple tissues and engages in numerous signal transduction pathways [61,62]. Ptpn1 contributes significantly to insulin signaling by dephosphorylating the insulin receptor and the insulin receptor substrate (IRS-1). A previous finding using knockout mice revealed the link of Ptpn1 to adiposity. Hence, Ptpn1 deletion appears to confer resistance against weight gain [61,63]. In pre-adipose cells, an almost similar reduction in B2m expression (~12-fold) was induced by both extracts. Meanwhile, Southern Home extract inhibited Sort1. B2m, the crucial gene is considered a biomarker in different diseases. According to the previously established study, an elevated level of serum B2m is associated with various adverse health effects, including overweight/obesity [64]. The gene Sort1was significantly attenuated (−9-fold). This gene is crucial in managing the metabolic phenotype, as revealed by a previous in-vivo study [65]. However, when they received the same diet, mice with diminished levels of Sort1 showed an overall lower body weight change and less visceral fat compared to their counterpart W.T.-type mice. Furthermore, insulin tolerance tests highlighted the boosted glucose uptake [65,66].

Interestingly, the impact of the two extracts under investigation on Hsp90ab1 expression was differently shown. This gene was downregulated in Southern Home-treated pre-adipose cells. Meanwhile, a similar reduction was measured in pineapple-treated adipocyte cells. Hsp90ab1 plays an essential role in the metabolic pathway [67]. Knockdown Hsp90ab1 in the mouse model of diabetes demonstrated the key role of this gene in controlling diabetes-mediated signaling pathways such as glucose metabolism and insulin signaling [68]. In parallel, both extracts inhibited the expression of Nr3c1 in pre-adipose cells by ~2–3-fold. However, the same reduction was exclusively found in Southern Home extract-treated adipocyte cells. Obesity, hyperinsulinemia, and abdominal visceral fat are associated with Nr3c1. Treatment of bipolar disorder with MS and SGAP is associated with significant side effects, including weight gain.

Furthermore, Nr3c1 is significantly associated with abdominal obesity [69,70,71]. In adipocyte cells, the Southern Home extracts inhibited the expression of two glucose homeostasis regulators: Insr and Il6ra. Insr is expressed mainly in insulin-sensitive tissues [72]. Skeletal muscle incorporates high expression of the Insr-b variant that is closely associated with insulin resistance and type 2 diabetes [73]. Consistently, an upregulated level of Insr-B was also found in adipocytes in people with type 2 diabetes compared with those with normoglycemic [74]. Upon binding, Il6ra is a crucial factor in Il-6 release [75]. In rodents and humans, a positive correlation was revealed between Il-6 levels and increased fat mass [76], with levels decreasing in weight loss [77]. The downregulated levels obtained in this study support the role of Southern Home extract in managing obesity. The data also showed an inverse effect of the two extracts under investigation. In pre-adipose 3T3-L1 cells, Pineapple induced a significant downregulation of Npy1r. In contrast, the same genes were upregulated in Southern Home-treated cells. Upregulated expression of Npy1r [78] was previously found in different cell models, such as human adipose cells, preadipocytes, and adipocytes 3T3-L1 cells [79,80,81]. Consistent with this observation, elevated levels of Npy1r were also measured in obese patients [82]. Having Npy1r downregulated by Pineapple extract suggests its potential role in managing obesity.

## 5. Conclusions

The current research provides insights into the mechanism underlying the anti-obesity potential of the Muscadine grape berry extracts (Pineapple and Southern Home) in both preadipocytes and adipocytes 3T3-L1. Our finding demonstrated the potential of the extracts under investigation to alter the expression of various genes regulating insulin and energy homeostasis. The most noteworthy finding of the current study is the outstanding upregulation of two genes, Cntfr and Hrh1, that were measured in Pineapple extract-treated adipocytes, in addition to the high fold-increase in Ramp3 induced by both Pineapple and Southern Home in preadipose cells. Both extracts showed a similar pattern of gene alteration, including the downregulation of B2m and Nr3c1 in preadipose cells and the inhibition of Ptpn1 and Adipore1 in adipocytes. Southern Home extract also showed a potential to inhibit other genes, including Sort1 in preadipose cells, Insr, and Il6ra in adipocytes. A downregulation of Hsp90ab1 was also shown in Southern Home-treated preadipose cells, and Pineapple-treated differentiated cells. In preadipocytes, the two extracts showed an inverted regulation of Npy1r, giving a mild fold increase in Southern Home treated and downregulation in Pineapple-treated cells.

Interestingly, pre-adipocyte-treated cells exhibited an increase in Atrn. However, the same gene was exclusively inhibited in Southern Home-treated adipocytes. Indeed, the obtained data suggested the antiobesity potential of Muscadine grape berry extracts (Pineapple and Southern Home). However, we plan to investigate these extracts further using an in-vivo model and fractionated extracts to identify the active components and fulfill the current research’s limitations.

## Figures and Tables

**Table 1:** A comparative illustration of Pineapple and Southern Home extracts impacts mRNA gene regulation in pre-adipose 3T3-L1 mouse cells.

Impacted Genes	Pineapple		Southern Home	
	Fold Change	*p*-Value	Fold Change	*p*-Value
Ramp3	+44.87	0.0107	+174.24	0.0009
Atrn	+2.36	0.0019	+1.67	0.0067
B2m	−13.59	0.0228	−12.20	0.0226
Sort1			−9.00	0.0067
Hsp90ab1			−2.62	0.0047
Nr3c1	−2.25	0.0248	−2.98	0.0084
Npy1r	−1.70	0.0127	+1.85	0.0076

**Table 2:** A comparative illustration of Pineapple and Southern Home extracts’ impacts on mRNA gene regulation in differentiated 3T3-L1 mouse cells.

Impacted Genes	Pineapple		Southern Home	
	Fold Change	*p*-Value	Fold Change	*p*-Value
Cntfr	+712.715	<0.0001		
Hrh1	+270.11	0.0187		
Zfp91	+7.90	0.0050		
Adipor1	−9.58	0.0163	−2.10	0.0287
Hsp90ab1	−3.40	0.0228		
Insr			−3.61	0.0037
Nr3c1			−2.97	0.0006
Ptpn1	−2.01	0.0261	−5.90	0.0015
Atrn			−3.30	0.0121
Il6ra			−4.50	0.0300

## Data Availability

The original contributions presented in the study are included in the article, further inquiries can be directed to the corresponding authors.
